# Supplementary material for: Patterns of Intron Gain and Loss in Fungi
Source: PLoS Biol. 2004 Nov 30;2(12):e422. doi: 10.1371/journal.pbio.0020422 (PMC532390; doi:10.1371/journal.pbio.0020422)
Supplement: Table S1 — Also available at http://genes.mit.edu/NielsenEtAl/. (4.3 MB ZIP). [file pbio.0020422.st001.zip › NielsenEtAl/html/1233.html]

AN1199.1.NCU08880.1.MG06782.1.FG10375.1


```
 CLUSTAL W (1.82) Multiple Sequence Alignments - Introns Inserted


Sequence 1: NCU08880.1	520 aa
Sequence 2: MG06782.1	532 aa
Sequence 3: FG10375.1	515 aa
Sequence 4: AN1199.1	497 aa
Alignment Length: 537 aa
Number Identitical Residues: 233 aa
Alignment Score (without introns) 11616


MG06782.1 	MSKESNRDVSPDGLTSS-PPDIQQNGGNGTQLR-TPLRDFRQWFG--GPGVTIGPRITSV
NCU08880.1	MPAPTPDPPRDNGREKSRIDDATASTACKEDVRPSSLWSWTKLDDTTAVGPRIGPVLQSV
FG10375.1 	MAEKR----SVGSGSQSPDPERRQSQVQIPSSRWASLFGG----AGVTVGPRIAQLPEFV
AN1199.1  	MAGFPLFG-TAREQSRSPSSDSSADKLKDKDLERSPQLAG--------------------
          	*.              *   :   .     . . :.                        

MG06782.1 	VVPSVESDSEAGSELVQKQIDSEANAGIQYRTCSWQK0TAALLFSEYICLAIMSFPWSYS
NCU08880.1	ATASSDSDDSIASPERQRELE-KGNA-IQYRTCSWQK0TAALLFSEYICLAIMSFPWSYS
FG10375.1 	RRDISDSEDSS-SAILNKQLQAEQGEQIQYRTCSWQK~TAGLLFSEYICLAIMSFPWSYS
AN1199.1  	--GLSDSDSGE----IGRQIELEAENSIKYRTCSWQK0TAALLFSEYICLAIMSFPWSYS
          	     :*:.        :::: :    *:******** **.*******************

MG06782.1 	ILGLVPG2LIFTVFVALVVLYT2SLVLW~EFCLRHPEVRDVCDIGQMLFWGKKWAWYATA
NCU08880.1	VLGLVPG2--------------~SLVLW~EFCLRHPEVRDVCDIGQMLFWNKNWAWWATA
FG10375.1 	VLGLVPG~IILTIVIALIVLYT~SLVLW~EFCLRHPEMRDVCDIGQMLFWDKKWAWWATA
AN1199.1  	ILGLVPG~LILTVFIAGVVLYT~SLIVW2RFCLRHPHIRDVCDIGQHLFWDSKIAWYFTA
          	:******    :   :     : **::* .******.:******** ***..: **: **

MG06782.1 	VMFLLNNT0FIQ0GLHVLVGAKYLNTMTGRSDSVQCQTVSFGIIVAIVCWLCSLPRTFSM
NCU08880.1	VMFLLNNT0FIQ~GLHVVVGAEYLNTMTGTHPGIACRTVSFGIIIALVCWLCSLPRTFNT
FG10375.1 	VCFILNNT~FIQ~GLHVLIGAKYLNTMTESDDIGHCRTVMFSAVVAIISWVCSLPRTFDM
AN1199.1  	VMFLLNNT~FIQ0GLHCVVGAEYLNTISNHG----TCTVVFSFVVAVISLVCSIPRTFGT
          	* *:**** *** *** ::**:****::         ** *. ::*::. :**:****. 

MG06782.1 	LSKLGTASAAFTFISVLLATIFAGIQGDPAGFDPRDSYTTPAGVAKVGGNPIVTAFPTAS
NCU08880.1	LSKLGVFSALFTFISVVLAAAFSGAQKHPAGYDPRPSFTSDSGVASVGGNPIVTALPVKS
FG10375.1 	MAKLGTASAFFTFISVLLAAIFAGIQAHPFGYDP----------AKLG-EPIVTAIPVKG
AN1199.1  	LAKVATISALATFVSVMLAVIFTAIEDHPARYNS---------TPEYSGEPIVKAFPVPG
          	::*:.. **  **:**:**. *:. : .*  ::.          .. ..:***.*:*. .

MG06782.1 	TTFVMATTAFLNISYTFIGQITLPSFIAEMKDPR2DFPKALWACTIAEIFVFSIVGSVIY
NCU08880.1	ASYIACFNAFLNISYTFIGQITLPSFIAEMREPK2DFPKALWACAIAEFILFSVVGGVIY
FG10375.1 	TTFVNGMSAFLNISYTFIGQITLPSFIAEMRDPR~DFPKALWACTIAEIIVFSLVGAIVY
AN1199.1  	TTFVSGMSAFLNISYTFIGQITLPSFIAEMREPK~EFWKSVTAVTIAEIIVFSLVGAIVY
          	::::   .**********************::*: :* *:: * :***:::**:**.::*

MG06782.1 	AYTGNQYMTSPAFGSL-HDVYKKVSFSFMIPTIIFLGVLYASVSARFIFFRLFDGSRHKS
NCU08880.1	GYTGNQYMTAPAFGSL-QETYKKISFSFMIPTIIFLGVLYASVSTRFIFFRIFKNSQHMT
FG10375.1 	NYTGNQYVTAPAFGSL-EDLYKKVAFSFMIPTIIFLGCLYASVSARFVFFRLFRNSKHLN
AN1199.1  	AYTGNQYMAAPAFGAIGDEVYKKVSFSFMIPTIIFLGVLYASVSARFIFFRLFEGTRHKG
          	 ******:::****::..: ***::************ ******:**:***:* .::*  

MG06782.1 	EHTVLGWSAWAAILF1TTWILAFIVAEVIPFFSDL1LSLMSSLFDSFFGFIFWGVAYFRM
NCU08880.1	EHTLTGWSTWAGILL1TTWICAWIIAEVIPFFSSL1LSLMSSLFDSFFGFIFWGVAYFRM
FG10375.1 	SHTIVGWGSWSGILL~VTWVFAFIIAMIIPFFNSL1LSVMSSLFDSWFGFIFWGVAYFRM
AN1199.1  	NHTFVGWASWIGILA~ALWAAAFIIAEVIPFFSDL~LSIMSSLFDSFFGFIFWGVAYLRM
          	.**. **.:* .**  . *  *:*:* :****..* **:*******:**********:**

MG06782.1 	RRADRRKNPDEQQTATGIALSALNVFIILIGVFFLTVGTY~ATVQSIIDSFHLGAVGTVF
NCU08880.1	RKADARAGITRKRGIGTILLDGTNIIIILVGFLFLSFGSY~ASVLGILEAYDTGSVRSVF
FG10375.1 	RTADKEIG-RHHSKVVDLAGNILNLVLIAIGIVFLTVGTY~ASVQGIIDQYAAGTVSGVF
AN1199.1  	RYDDHGPGFYRNRGFRGWVGAIVNVGLIGIGLYFLGPGTY0ASVESVIIDYRAGSFGGPF
          	*  *   .  .:           *: :* :*. **  *:* *:* .::  :  *:.   *

MG06782.1 	TCASNAL
NCU08880.1	SCASNGL
FG10375.1 	SCASNGL
AN1199.1  	TCADNGL
          	:**.*.*
```
